# Planned Relook Laparotomy for the Salvage of Extensive Small Bowel Ischemia in Primary Volvulus: A Case Report From a Resource-Limited Setting

**DOI:** 10.7759/cureus.97185

**Published:** 2025-11-18

**Authors:** Yonas M Nur, Yoseph M Habte, Binyam M Habte, Esimael M Abdu, Makida M Habte

**Affiliations:** 1 Department of Surgery, University of Gondar, Gondar, ETH; 2 Department of Medicine, Ethio Tebib Hospital, Addis Ababa, ETH; 3 Department of Medicine, ALERT Comprehensive Specialized Hospital, Addis Ababa, ETH; 4 Department of Surgery, Teklehaimanot General Hospital, Addis Ababa, ETH; 5 Department of Medicine, Bethel Medical College, Addis Ababa, ETH

**Keywords:** case report, primary small bowel volvulus, relook laparotomy, resource-limited setting, small bowel ischemia

## Abstract

Extensive small bowel ischemia due to volvulus poses a major surgical challenge: premature resection risks short bowel syndrome, while delayed intervention can lead to necrosis and sepsis. We report the successful salvage of extensive small bowel ischemia using a staged surgical approach with planned relook laparotomy in a 34-year-old woman who presented with recurrent small bowel obstruction. Intraoperatively, a 360° volvulus extending from 10 cm distal to the ligament of Treitz to 2 cm proximal to the ileocecal valve was identified, with diffuse ischemia. After derotation and warm saline application, partial reperfusion was observed, and immediate resection was deferred. A planned relook laparotomy 48 hours later revealed complete recovery of bowel viability. Despite the favorable surgical outcome, the patient succumbed on postoperative day 26 to hospital-acquired pneumonia, unrelated to the primary surgical pathology. This case underscores the importance of individualized intraoperative decision-making and supports planned relook laparotomy as a safe strategy to restore marginally ischemic bowel and prevent unnecessary resection, particularly in resource-limited settings.

## Introduction

Small bowel obstruction (SBO) remains a major cause of acute surgical abdomen, with approximately 350,000 cases reported annually in the United States [1]. In Ethiopia, the prevalence of intestinal obstruction among patients presenting with acute abdomen ranges from 18.6% to 50.7% [2]. Primary small bowel volvulus (SBV) is a rare etiology of SBO in adults but can rapidly progress to ischemia and bowel necrosis if not promptly diagnosed and managed [3].

Diagnostic imaging plays a crucial role in confirming the diagnosis, determining the level and cause of obstruction, and identifying associated complications such as ischemia, necrosis, or perforation [4,5]. Intestinal ischemia is a potentially fatal condition owing to its non-specific clinical presentation, necessitating a high index of suspicion for timely intervention [6]. When small bowel ischemia is suspected or evident, exploratory laparotomy becomes essential.

Traditionally, management involves resection of the non-viable bowel segment with primary anastomosis [7]. However, given the potential morbidity associated with extensive resection and the risk of developing short bowel syndrome (SBS), a more conservative strategy may be considered in selected patients. In cases where bowel viability is uncertain, a staged approach with planned relook laparotomy allows reassessment of intestinal viability and the possibility of salvaging borderline segments, particularly if intervention occurs within the critical ischemic window, typically within six hours of onset [8].

This case report presents the management of a 34-year-old woman with extensive ischemic small bowel secondary to primary volvulus. A staged surgical approach with planned relook laparotomy successfully restored bowel viability and preserved intestinal length. Although the patient later succumbed to hospital-acquired pneumonia unrelated to the primary pathology, the case highlights the value of relook laparotomy in selected patients with potentially reversible small bowel ischemia.

## Case presentation

A 34-year-old Ethiopian woman presented with severe, crampy periumbilical pain lasting three hours, accompanied by non-projectile bilious vomiting and abdominal distension. She denied fever, constipation, or obstipation. Four weeks earlier, she had undergone laparotomy and derotation for primary SBV and was readmitted four weeks later for adhesive SBO.

On examination, the patient appeared acutely ill but hemodynamically stable (BP 110/70 mmHg, pulse 68 bpm, respiratory rate (RR) 20/min, temperature 36°C, SpO₂ 97% on room air). The abdomen was markedly distended and tense, with hyperresonance on percussion and a midline laparotomy scar. There was no tenderness or peritoneal sign.

Laboratory findings revealed leukocytosis (white blood cell (WBC) 16,400/µL, 80% neutrophils) and mild hypokalemia. Other biochemical parameters were within normal limits (Table 1).

**Table 1:** Laboratory investigations with corresponding results and reference values on initial presentation

Laboratory parameters	Results	Normal value
Complete blood count		
White blood cell	16.4 × 10^3^/µL	4.0-11.0 × 10^3^/µL
Hemoglobin	14.2 g/dL	12.5-15.5 g/dL
Platelet	402 × 10^3^/µL	150-450 × 10^3^/µL
Lymphocyte percentage	16%	15%-50%
Neutrophil percentage	80%	45%-80%
Metabolic panel		
Creatinine	0.8 mg/dL	0.5-1.1 mg/dL
Urea	17.9 mg/dL	17-43 mg/dL
Na^+^	139 mmol/L	136-145 mmol/L
K^+^	3.04 mmol/L	3.5-5.1 mmol/L
Aspartate transaminase	40.1 U/L	2-50 U/L
Alanine transaminase	23.6 U/L	1-50 U/L
Alkaline phosphatase	174 U/L	70-260 U/L
C-reactive protein	3.35 mg/L	<5 mg/L

**Figure 1:**
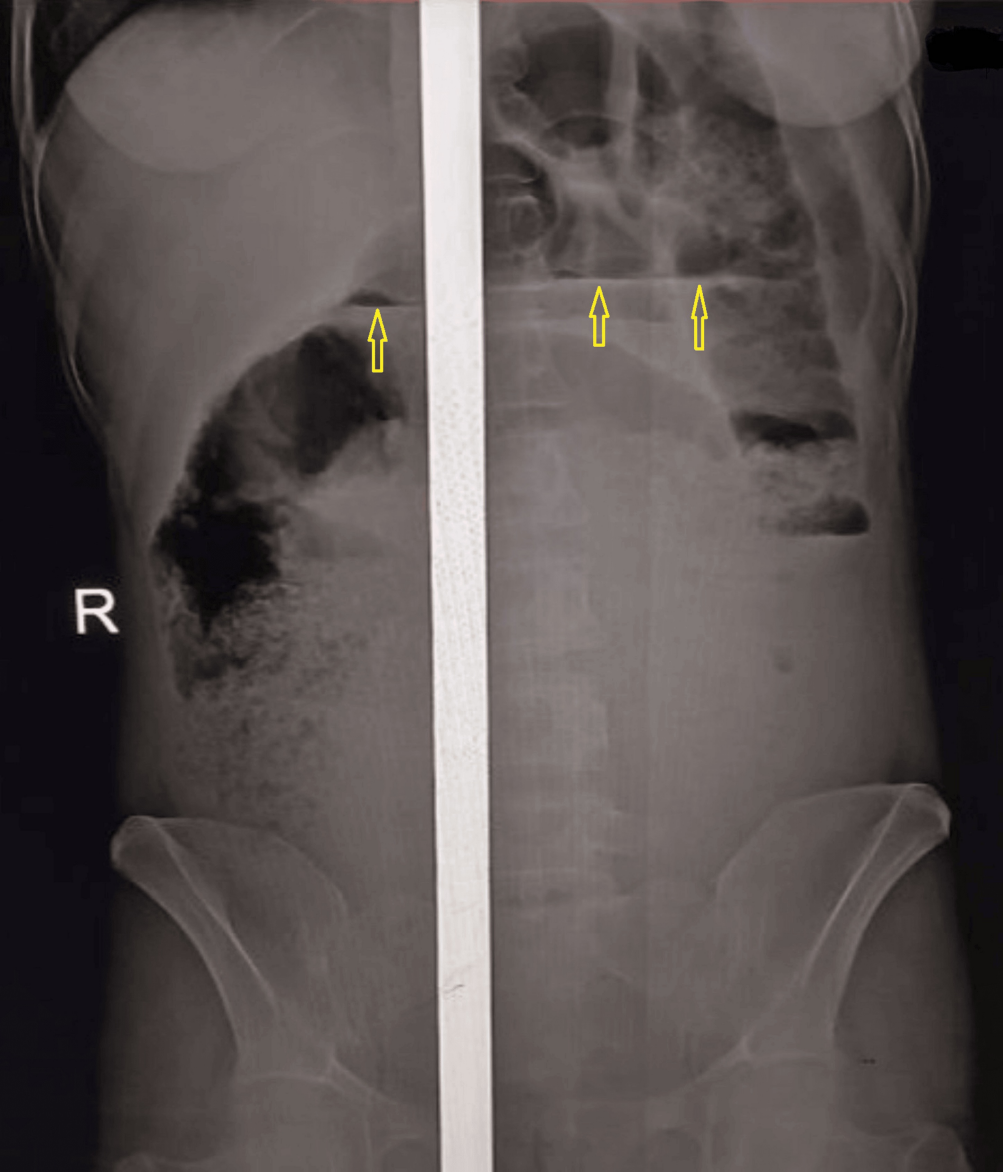
Erect abdominal X-ray showing small bowel obstruction Multiple dilated small bowel loops with air-fluid levels (yellow arrows) are visible, consistent with small bowel obstruction. The vertical white line in the center represents a cassette or detector artifact from an older X-ray system still in use in our hospital and has no diagnostic significance. The image has been preserved in its original, unaltered form to maintain anatomical integrity.

An erect abdominal X-ray showed multiple air-fluid levels and dilated small bowel loops, consistent with SBO (Figure 1).

**Figure 2:**
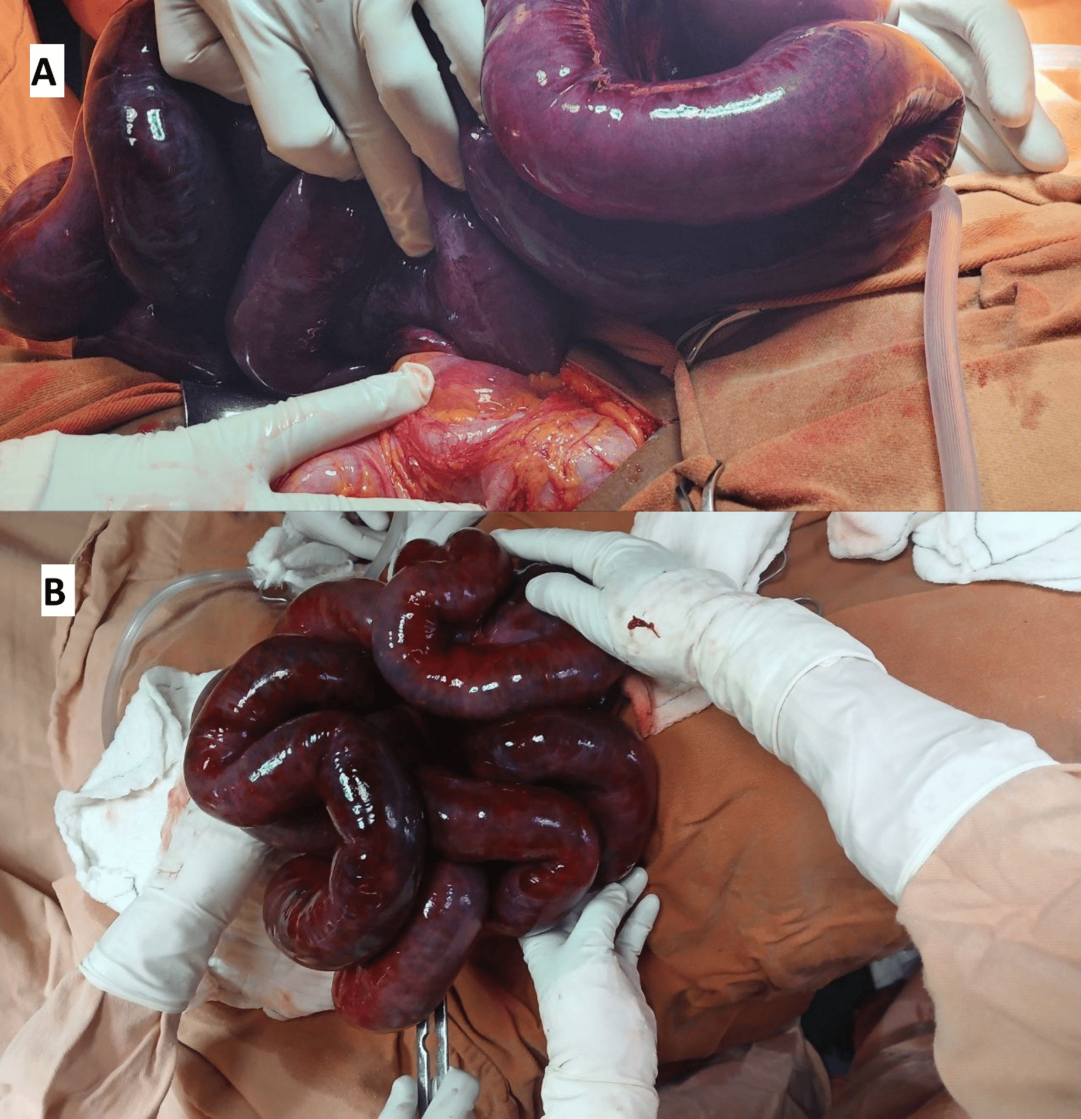
Grossly visible intraoperative findings at the initial laparotomy (A) Small bowel rotated 360°. (B) Ischemic, purplish-dusky segment of the small intestine.

Despite fluid resuscitation and nasogastric decompression, the patient’s abdominal pain worsened. Her vital signs deteriorated within 1.5 hours of admission, prompting urgent exploratory laparotomy. A midline incision revealed 500 mL of hemorrhagic peritoneal fluid and a 360° SBV extending from 10 cm distal to the ligament of Treitz to 2 cm proximal to the ileocecal valve. The entire volvulated segment appeared dusky and non-perfused (Figure 2). Limited reperfusion was observed in a small segment after 20 minutes of warm saline application. Given the inadequate residual bowel length for resection, the abdomen was irrigated, decompressed via two enterotomies, and temporarily closed for reassessment within 48 hours.

**Figure 3:**
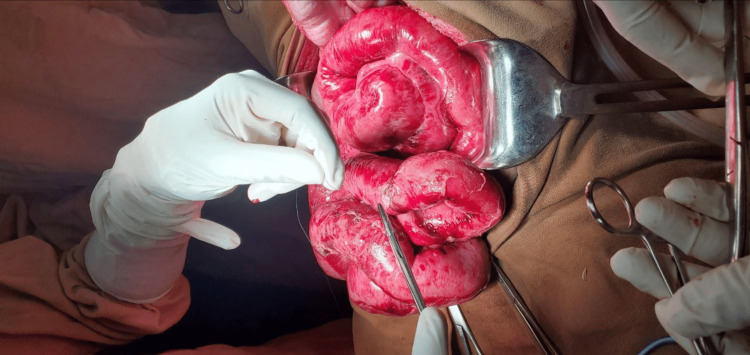
Intraoperative findings at the planned relook laparotomy The entire small bowel appears pink, viable, and peristaltic.

At the planned relook laparotomy 48 hours later, 100 mL of reactive peritoneal fluid was noted, and the entire small bowel appeared pink, viable, and peristaltic (Figure 3). The enterotomy sites were imbricated, the peritoneal cavity was washed with saline, and the abdomen was closed with retention sutures.

The postoperative course was initially uneventful until day three, when the patient developed fever, cough, dyspnea, and hypoxia. She was diagnosed with severe hospital-acquired pneumonia requiring immediate ventilatory support, broad-spectrum intravenous antibiotics, and intensive care. Despite these interventions, her condition rapidly deteriorated, resulting in refractory respiratory failure. She died on postoperative day 26 from severe pneumonia, unrelated to the primary surgical pathology, as there was no evidence of intra-abdominal sepsis, anastomotic leakage, or wound infection on clinical and laboratory assessment.

## Discussion

Adult-onset SBV is a rare cause of acute abdominal pain that can rapidly progress to bowel ischemia if not promptly managed. SBV may arise from congenital, primary, or secondary etiologies [3]. In this case, a 34-year-old woman presented with recurrent SBO six weeks after an initial laparotomy and derotation for primary SBV. She had experienced postoperative adhesions four weeks prior, managed conservatively, and now presented with a rare recurrence of primary SBV leading to extensive intestinal ischemia.

Classic clinical features of SBO include abdominal pain, nausea, vomiting, abdominal distension, and constipation progressing to obstipation. Physical findings range from restlessness and dehydration to signs of sepsis, such as tachycardia, fever, hypotension, or orthostatic changes. Abdominal distension and hypoactive bowel sounds are common, while severe tenderness, rebound tenderness, rigidity, or guarding suggest advanced SBO [1]. Our patient presented with severe, crampy, intermittent periumbilical pain, vomiting, and abdominal distension. Examination revealed a grossly distended, tense, non-tender abdomen with marked tympany and a midline surgical scar. Vital signs were initially stable but subsequently deteriorated, with pulse rate increasing from 68 to 90 bpm.

Laboratory findings in SBO often include mild leukocytosis and electrolyte imbalances due to dehydration and third-space fluid shifts [9]. Elevated lactate and acidosis may indicate intestinal strangulation [10]. Imaging is critical for diagnosis: plain abdominal radiographs typically show dilated small bowel loops and multiple air-fluid levels, while CT imaging can detect signs of strangulation, including reduced bowel wall enhancement, mesenteric fluid, and pneumatosis [11]. In this case, laboratory results demonstrated leukocytosis (WBC 16,400/µL with 80% neutrophils) and mild hypokalemia; other organ function tests were normal. An erect abdominal radiograph confirmed SBO; CT imaging and serum lactate were unavailable.

Surgical intervention is indicated in SBO when there are signs of generalized peritonitis, clinical deterioration, fever, leukocytosis, tachycardia, metabolic acidosis, or persistent severe pain [12]. Early surgery is critical: intestinal salvage rates remain around 80% if performed within 200 minutes of diagnosis but drop below 50% if delayed beyond 300 minutes [13,14]. Physiologic consequences of ischemia are generally reversible within six hours from symptom onset [14].

In our patient, deteriorating clinical status necessitated an exploratory laparotomy. Intraoperatively, a large segment of the small intestine, from 10 cm distal to the ligament of Treitz to 2 cm proximal to the ileocecal valve, appeared ischemic. Standard management involves resection of the ischemic segment followed by primary anastomosis [7]. Immediate resection was deferred to avoid SBS, a condition caused by insufficient intestinal length leading to malnutrition and complex nutritional management [15]. Given her presentation within 4.5 hours and the presence of partial bowel viability, a planned relook laparotomy was performed 48 hours later. This was based on intraoperative clinical judgment, allowing sufficient time for borderline bowel to declare its viability while minimizing the risk of progression to necrosis.

This case highlights several important clinical lessons. Adult-onset SBV, though rare, should be considered in patients with acute abdominal pain and recurrent SBO. Early surgical assessment and timely intervention are essential to prevent irreversible ischemia. Planned relook laparotomy can allow marginally ischemic bowel to recover, minimizing the risk of SBS. Despite a favorable surgical outcome, the patient ultimately succumbed to severe hospital-acquired pneumonia, resulting in respiratory failure unrelated to the primary surgical pathology.

## Conclusions

This case underscores the importance of early surgical intervention and individualized intraoperative decision-making in the management of extensive small bowel ischemia. Staged reassessment through a planned relook laparotomy can allow marginally ischemic bowel to recover, avoiding immediate resection and the complications of SBS. In carefully selected patients, this approach can optimize bowel preservation, improve postoperative outcomes, and highlight the potential benefits of tailored surgical strategies in complex acute abdominal emergencies. Wider adoption of this strategy may guide the management of borderline ischemic bowel in other patients, potentially reducing unnecessary resections and associated morbidity.
